# The Neural Association Between Symptom and Cognition in Major Depressive Disorder: A Network Control Theory Study

**DOI:** 10.1002/hbm.70198

**Published:** 2025-03-20

**Authors:** Aoxiang Zhang, Qian Zhang, Ziyuan Zhao, Qian Li, Fei Li, Yongbo Hu, Xiaoqi Huang, Weihong Kuang, Graham J. Kemp, Youjin Zhao, Qiyong Gong

**Affiliations:** ^1^ Department of Radiology, Huaxi MR Research Center (HMRRC), Institute of Radiology West China Hospital of Sichuan University Chengdu Sichuan China; ^2^ Research Unit of Psychoradiology Chinese Academy of Medical Sciences Chengdu Sichuan China; ^3^ Functional and Molecular Imaging Key Laboratory of Sichuan Province West China Hospital of Sichuan University Chengdu Sichuan China; ^4^ Department of Psychiatry West China Hospital of Sichuan University Chengdu China; ^5^ Liverpool Magnetic Resonance Imaging Centre (LiMRIC) and Institute of Life Course and Medical Sciences University of Liverpool Liverpool UK; ^6^ Xiamen Key Laboratory of Psychoradiology and Neuromodulation, Department of Radiology West China Xiamen Hospital of Sichuan University Xiamen Fujian China

**Keywords:** cognition, depression, major depressive disorder, network controllability, psychoradiology

## Abstract

Major depressive disorder (MDD) is characterized by intercorrelated clinical symptoms and cognitive deficits, whose neural mechanisms in relation to these disturbances remain unclear. To elucidate this, we applied the relatively new approach of Network Control Theory (NCT), which considers how network topology informs brain dynamics based on white matter connectivity data. We used the NCT parameter of average controllability (AC) to assess the potential control that brain network nodes have on brain‐state transitions associated with clinical and cognitive symptoms in MDD. DTI and high‐resolution T1‐weighted anatomical imaging were performed on 170 MDD patients (mean age 31.6 years; 72 males, 98 females) and 137 healthy controls (HC; mean age 33.4 years; 64 males, 73 females). We used an NCT approach to compare AC between the groups. We then performed partial Spearman's rank correlation and moderation/mediation analyses for AC and cognition and clinical symptom scores. Compared with HC, MDD patients had lower AC in the left precuneus and superior parietal lobule and higher AC in the right precentral gyrus (preCG) and superior frontal gyrus (SFG), predominantly in the default‐mode, somatomotor, and attention networks. In the HC group, AC of right preCG was positively associated with processing speed. While in the MDD group, AC of right SFG was negatively associated with memory function and also negatively moderated the association between memory and anxiety symptoms. The current study highlighted that the altered brain controllability may provide a novel understanding of the neural substrate underlying cognitive control in MDD. Disrupted control of right SFG during state transitions may partially explain the variable relationship between memory and anxiety symptoms in MDD.

## Graphical abstract

We aimed to determine the effect of alterations in average controllability (AC) on emotional and cognitive symptoms in patients with major depressive disorder (MDD). We found that AC values in the right SFG were negatively moderating the association between memory (i.e., DSF scores) and anxiety symptoms (i.e., HAMA scores) in patients with MDD.

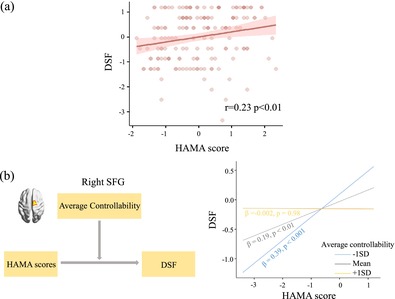

**Summary**

- Altered brain average controllability (AC) in regions of the default mode, somatomotor, and attention networks was identified in patients with major depressive disorder (MDD) compared to healthy controls (HC).
- Patterns of controllability–cognition correlation differed between MDD and HC, which may reflect the impact of white matter pathway damage on typical cognition associations in MDD patients.
- The AC of the right superior frontal gyrus negatively moderated the relationship between memory and anxiety symptoms, highlighting its potential role in the interplay between cognitive and emotional disturbances in MDD.
- These findings offer neuroimaging biomarkers for targeted interventions that may potentially modulate functional outcomes in MDD.

## 1 Introduction

Major depressive disorder (MDD) is characterized by both clinical symptoms and cognitive impairments (Gong and He 2015; Gotlib and Joormann 2010; Otte et al. 2016); these are common and often coexist, and are at least partly treatable (Tam and Lam 2012). However, their relationship is not clear: worse depression symptoms are reportedly associated with worse cognitive dysfunction (Matcham et al. 2023; Tam and Lam 2012; Withall et al. 2009), but this has not always been replicated (Kyte et al. 2005; Milders et al. 2010; Reppermund et al. 2009). Why the severity of symptoms in MDD has such a variable impact on cognition remains to be fully explained. Heterogeneity of methodology and patients is no doubt important (McDermott and Ebmeier 2009). However, there may also be moderating factors that influence cognitive function in MDD (Du et al. 2022; Park et al. 2018; Phillips et al. 2015). Additionally, unbalanced symptom–cognition interactions may disrupt adaptive behavior in depression, but the underlying mechanism is not fully understood (Dolcos et al. 2011; Liang et al. 2023; Snyder 2013).

Like many neuropsychiatric disorders, MDD is increasingly conceptualized as a brain disconnection disorder (Otte et al. 2016). Studies using diffusion tensor imaging (DTI) in MDD have reported alterations in the white matter (WM) structural connectome which are associated with both clinical symptoms and cognitive deficits. For instance, the WM connection between the left thalamus and postcentral gyrus is negatively correlated with the severity of depression (Lu et al. 2017). The fractional anisotropy (FA) of the anterior callosal tracts is reduced in patients with MDD, which may be related to the impairment of working memory and attention (Yamada et al. 2015). The nodal efficiency of the right dorsolateral superior frontal gyrus (SFG) is associated with executive functioning when FA is taken as the edge weight (Wang, Yuan, et al. 2020). This is therefore a promising framework for studying the complex relationship between structure and symptoms, which can explain the dynamic changes in the way cognition interacts with clinical symptoms as well as identify causal neural mechanisms mediating this interaction (Dolcos et al. 2011), and several methodological approaches are available (Li et al. 2021; Lui et al. 2016). DTI studies have traditionally examined pathways that directly connect regions, and more recently have explored network properties using graph‐theoretic measures (Korgaonkar et al. 2014; Rubinov and Sporns 2010; Sun et al. 2015; van Velzen et al. 2020). However, both approaches miss the impact of structural dysconnectivity on function, which requires consideration of the brain structure–function as a dynamic system (Dolan 2002). The recently developed approach of network control theory (NCT) attempts to integrate brain architecture and dynamics from a control perspective (Gu et al. 2015). This key concept is that external energy input from brain regions to brain networks modulates transitions from one brain state to another to meet changing functional demands (Gu et al. 2015). Critical to such transitions is the underlying structural connectome, which both restricts and facilitates dynamic brain function (Honey et al. 2009; Mišić et al. 2015; Uhlhaas and Singer 2012). An increasing number of studies have begun to investigate the relationship between structural connectivity and neural function (Hermundstad et al. 2013, 2014; Medaglia et al. 2018) and shown its value in inferring/predicting symptomatology from structural network alterations without direct functional data (Kenett et al. 2018).

In the NCT framework, a key metric is average controllability (AC), characterizing the efficiency with which a brain region can steer the brain system to target states (Gu et al. 2015). Graph‐based metrics provide a way to simplify complex systems into more straightforward representations to investigate network architecture (Sporns 2018). In contrast, AC metrics represent the ability of brain regions to change current functional states based on the underlying structural topology (e.g., transitioning from the executive state to the resting state or sustaining a particular state) (Tang et al. 2017). This system‐level property constitutes the main influence on the types of mental processes that can be supported (Lee et al. 2020; Mišić and Sporns 2016). NCT‐based studies have shown that AC increases throughout development (Cornblath et al. 2019; Tang et al. 2017) were related to cognitive functions (Cornblath et al. 2019; Cui et al. 2020), impulsivity (Cornblath et al. 2019) in healthy populations. NCT‐based studies on MDD have identified altered controllability in the default mode network (DMN), frontoparietal network (FPN), and dorsal attention network (DAN) in MDD patients, which further correlated with symptom severity, genetic, and familial risk in MDD (Fang et al. 2021; Hahn et al. 2023; Li et al. 2024). Altered controllability may impair attention, executive functions, and behavioral flexibility, potentially disrupting adaptive abilities and contributing to increased feelings of hopelessness and depression in MDD patients (Gotlib and Joormann 2010). As the transition between brain states is a critical part of cognitive control, exploring the underlying control mechanism and its interaction patterns with symptomatic and cognitive disturbances in MDD patients is essential.

In this study, we aimed to use NCT to examine the alterations in brain network controllability in first‐episode, drug‐naïve patients with MDD relative to healthy controls (HC) and explore the relationships between AC alterations, emotional symptoms, and cognitive function. We hypothesized that (1) brain AC would be altered in MDD patients, especially in DMN, FPN, and DAN; (2) these changes would be related to measures of symptom/cognition deficits; and (3) the association between symptom and cognition would be moderated or mediated by alterations in controllability.

## 2 Methods

### 2.1 Participants

Patients with MDD (*n* = 170) were recruited at West China Hospital from November 2010 to November 2018. Patients were diagnosed according to the Diagnostic and Statistical Manual of Mental Disorders, 4th edition (DSM‐IV) (First et al. 1997) and the inclusion criteria were as follows: age 18–65 years; first episode of MDD; no history of psychiatric medication; no psychiatric comorbidities, for example, anxiety disorders. HC (*n* = 137) were recruited by local advertisement, having no personal history or known history in first‐degree relatives of major physical or neurological illness. The exclusion criteria for all participants were significant systemic disorders, neurological illness, substance abuse or dependence, pregnancy, or MRI contraindications. This study was approved by the Ethics Committee on Biomedical Research, West China Hospital of Sichuan University, and written informed consent was obtained from all subjects.

The severity of depressive symptoms was assessed using the total score of the Hamilton Depression Rating Scale (HAMD, 17 items) (Hamilton 1960), while the severity of anxiety symptoms was evaluated using the total score of the Hamilton Anxiety Rating Scale (HAMA, 14 items) (Hamilton 1959). All MDD patients and (limited by organizational constraints) a subgroup of approximately half (*n* = 62) of the HC group recruited later in the study completed neuropsychological tests covering multiple cognitive domains, including memory, processing speed, and executive functioning. Short‐term memory was assessed using the Digit Span Forward (DSF) and Digit Span Backward (DSB) tests (Richardson 2007); the score for each was the number of accurate counts. Processing speed was assessed using the Trail‐Making Test (TMT) A and B (Salthouse 2011); the score for each item was the time to completion. Executive function was assessed using the Stroop Color‐Word Test (SCWT), with inhibition scores as the primary metric (Scarpina and Tagini 2017). All cognition score data were standardized by *z* scores to ensure scale consistency; negative *z* scores were used for TMT‐A/B and SCWT for consistency, such that all higher scores reflect better performance (Lin et al. 2020).

### 2.2 MRI Data Acquisition

MRI scans, including DTI and high‐resolution T1‐weighted anatomical imaging, were performed on a 3.0 T scanner (Siemens Trio). The DTI data were collected using a single‐shot echo‐planar imaging sequence with the following parameters: repetition time (TR) 6800 ms, echo time (TE) 93 ms, field of view (FOV) 230 × 230 mm^2^, voxel size 1.8 × 1.8 × 3.0 mm^3^, and slice thickness 3.0 mm with no interslice gap. Each DTI dataset included 40 noncollinear directions (*b* = 1000 s/mm^2^) and 2 additional images without diffusion weighting (*b* = 0 s/mm^2^). High‐resolution T1‐weighted images were acquired using a spoiled gradient recall sequence with the following parameters: TR 1900 ms, TE 2.26 ms, flip angle 9°, FOV 256 × 256 mm^2^, voxel size 1 × 1 × 1 mm^3^, slice thickness 1 mm with no gap, and 176 slices. The MR images were inspected by two experienced neuroradiologists (Zhang and Zhao) separately to exclude data with obvious brain abnormalities or ghosting artifacts.

### 2.3 DTI Preprocessing

DTI data processing was performed using the Pipeline for Analyzing Brain Diffusion Images toolkit (PANDA, http://www.nitrc.org/projects/panda) (Wang, Wang, et al. 2015) and included the following steps: data conversion from DICOM to NII; correction of head motion and eddy current; and calculation of voxelwise tensor matrix and diffusion tensor metrics (such as FA, the major measure of interest, which reflects WM integrity). We used the Lausanne 2008 atlas (Cammoun et al. 2012; Hagmann et al. 2008) (Table S1) to parcellate the brain into 220 cortical and 14 subcortical regions, each representing a node of the cortical network, in the DTI native space: each individual DTI image in native space was co‐registered to its corresponding T1‐weighted anatomical image using a linear transformation; the anatomical image was then nonlinearly mapped to the ICBM152 template; the resulting inverse transformation was then used to warp the Lausanne 2008 atlas from the standard space to the individual native space. Performing fiber assignment via a continuous tracking algorithm, we obtained WM deterministic fiber tracking with an angular threshold of 45° and a FA threshold of 0.2. We then calculated the FA values between the end nodes as connectivity weights and generated a weighted, symmetrical anatomical 234 × 234 matrix for each participant. Finally, 220 cortical nodes were examined, belonging to 7 canonical networks (Yeo et al. 2011): the DMN, FPN, DAN, ventral attention network (VAN), limbic network (LN), somatomotor network (SMN), and visual network (VN). As before (Baum et al. 2017), the 220 cortical nodes from the Lausanne atlas were assigned to Yeo's 7 functional networks based on the maximum voxel‐wise overlap. Each node was then assigned to the network with the highest overlap. Since the Yeo atlas does not include subcortical regions, 14 subcortical nodes in our study were assigned to an eighth “subcortical” network (Baum et al. 2017).

### 2.4 Network Controllability Analysis

Brain control refers to the alteration of regional brain activity generated by real‐time neurofeedback (Gu et al. 2015). In control theory, the controllability of a brain region is associated with its structural connectivity characteristics, which can facilitate or restrict transitions between various brain states (Gu et al. 2015; Lee et al. 2020). Thus, the WM controllability metric can predict each brain region's ability to drive the brain system from any initial state to a specific target state (Tang et al. 2017).

According to the well‐established application of structural NCT to the brain (Gu et al. 2015), we make the simplifying assumption that neural states follow a noise‐free time‐invariant linear model:

$$x\left(t+1\right)=\mathrm{Ax}\left(t\right)+B_{K}u_{K}\left(t\right)$$

where the vector $x\left(t\right)$ represents the state of all 234 Lausanne Atlas brain regions at time *t*, the symmetric adjacency matrix A indexes structural connections between each pair of regions, $K=\left\{k_{1},\ldots k_{m}\right\}\subseteq V$ is the set of control nodes, and the input matrix is $B_{K}=\left[e_{k_{1}}\ldots e_{k_{m}}\right]$ where $e_{i}$ is the *i*th canonical vector of dimension *N*. We computed AC for each brain region separately, for which $B_{K}$ simplifies to an *N* × 1 vector where the element corresponding to the region assessed is set to 1 and all other elements are set to 0, and $u_{K}\left(t\right)$ becomes an *N* × 1 vector describing the external energy input function at a given moment, that is, the control strategy.

In NCT, the controllability of a dynamical system relates to the possibility of driving its present state to a target state by means of an external control input (Gu et al. 2015). In this application we make use of the controllability matrix $C=\left[\mathrm{BAB}A^{2}B\ldots A^{n-1}B\right]$; the network is controllable when this is of full rank, that is, $\mathrm{rank}\left(C\right)=n$. The Gramian matrix W is defined to quantify the controllability of the network (Pasqualetti et al. 2019).

$$W=\sum_{\tau =0}^{T-1}A^{\tau }BB^{⊺}{\left(A^{⊺}\right)}^{\tau }=CC^{⊺}$$

Here, ⊺ denotes the matrix transpose, τ represents the trajectory's time step, and T is the time horizon, which is defined as an infinite value. W is computed separately for each node in A (Gu et al. 2015).

AC of a network is defined as the average input energy from a set of control nodes over all possible target states, and is proportional to $\text{Trace}\left(W^{-1}\right)$, the trace of the inverse of the controllability Gramian. For technical reasons it is preferable to use $\text{Trace}\left(W\right)$, to which $\text{Trace}\left(W^{-1}\right)$ has a relationship of inverse proportionality (Gu et al. 2015). To assess the AC of node *i*, we calculate $\text{Trace}\left(W\right)$ assuming that it is the only control node in the network.

### 2.5 Statistical Analyses

Since the controllability data of most brain regions were not normally distributed as assessed by the Kolmogorov–Smirnov test (Figure S2), group comparisons of AC were conducted by nonparametric permutation testing with 1000 iterations while controlling for age and sex. The between‐group difference in the mean AC value was calculated as the observed value of the test statistic. During each permutation, the HC and MDD groups were randomly shuffled, and the mean differences between the two permuted groups were recomputed. The proportion of the sampled permutations for which the mean differences were greater than the observed test statistic was determined as the *p* value. We used Cliff's Delta (ranges from −1 to 1) to estimate the effect size for group comparisons, as it is robust to non‐normal distributions and is not influenced by outliers (Macbeth et al. 2011). The false discovery rate (FDR) correction method for multiple comparisons was performed using the Benjamini‐Hochberg procedure. The significance threshold of *p* = 0.05 was adopted after FDR correction, with all brain regions considered (234 corrections). Finally, partial Spearman's rank correlation analyses were performed for cognition scores, AC, and clinical symptoms, with age and sex as covariates (see Supporting Information Material). All *p* values were corrected using the FDR method, considering the total number of correlation analyses conducted. The *F* test was used to determine the differential correlations between the groups, avoiding bias arising from the direct comparison of correlation coefficients when variances are unequal (Lenroot et al. 2007).

### 2.6 Comparison of Controllability Metrics With Traditional Graph‐Theoretic and White Matter Integrity Metrics

NCT emerged quite recently, and the utility of its perhaps rather abstract controllability measures in studying behavior is still being established. Nodal centrality (including nodal degree, nodal betweenness and nodal efficiency) metrics assess both the number of links a node has and its ability to propagate information across the network (Achard and Bullmore 2007; Rubinov and Sporns 2010). Previous evidence (Fang et al. 2021; Tang et al. 2023) has indicated the concurrence of abnormalities in both nodal centrality and controllability in mental disorders. They further found that the AC was positively correlated with nodal degree, nodal betweenness, and nodal efficiency. In other words, brain regions with higher AC tend to be highly connected hubs that transmit information efficiently, which may offer more insights into the pathophysiological implications of the MDD‐related controllability alterations. We further speculated that the brain regions with higher AC have more complete underlying WM pathways, thereby supporting dynamic brain transitions.

We first compared regional nodal centralities (nodal degree, nodal efficiency, and nodal betweenness) and WM integrity metric (i.e., FA, reflecting the integrity of WM tracts) in the two groups using permutation tests at the whole brain level. Notably, we mainly focused on the group differences in node centrality and FA metrics in brain regions with significant AC alterations. Second, in regions showing significant AC differences between MDD patients and HC, partial Spearman's rank correlation analyses were used to examine the associations of AC with nodal centrality metrics and FA in the MDD and HC groups. Age and sex were treated as covariates in the between‐group comparison and univariate correlation analysis, and findings were corrected with FDR to preserve a *p* < 0.05 threshold (see Supporting Information Methods for details).

### 2.7 Mediation and Moderation Analysis

We used PROCESS V4.2 for SPSS to explore the possible mediating or moderating effect of the AC indicator between cognition and emotional symptoms in MDD patients. We selected model 1 in PROCESS to test the significance of the moderating effect and model 4 to test the mediating effect. The model was set up as follows: the independent variable was HAMD/HAMA total score, the dependent variable was the cognitive score, and the moderator or mediator was the brain controllability metric AC, with sex and age as covariates. The 95% confidence intervals (CIs) of the moderation and mediation effects were calculated with a nonparametric bootstrap (5000 times). All –values of *p* < 0.05 were considered to be statistically significant with FDR correction for multiple moderation and mediation models.

## 3 Results

### 3.1 Participant Characteristics

Table 1 summarizes the demographic and clinical characteristics of the participants. The MDD group (*n* = 170, mean age 31.6 ± 11.5 years, 72 males and 98 females) and the whole HC group (*n* = 137, mean age 33.4 ± 10.4 years, 64 males and 73 females) did not significantly differ in age and gender. However, significant differences in gender (*p* = 0.03) were found between the MDD group and the later‐recruited subgroup of HC with available cognitive data (*n* = 62, mean age 33.4 ± 10.8 years, 36 males and 26 females). As for neuropsychological tests, the MDD group only had lower DSF scores than the HC subgroup (*p* = 0.03), with no significant group differences in the remaining variables.

**TABLE 1:** Demographic and clinical information of first‐episode drug‐naïve MDD patients and HC.

	MDD (*n* = 170)	HC (*n* = 137)	HC (*n* = 62)	*t* or *χ*2	*p* ^a^
Age (years)	31.6 ± 11.5	33.4 ± 10.4	33.4 ± 10.8	−1.49/−1.04	0.14/0.29
Sex (female/male numbers)	98/72	73/64	26/36	0.59/4.50	0.44/**0.03** ^b^
Duration of untreated disease (weeks)	35.9 ± 56.6	—	—	—	—
Clinical symptom severity scores					
HAMA‐14 total score	22.4 ± 9.1	—	—	—	—
HAMD‐17 total score	24.2 ± 6.3	—	—	—	—
Neuropsychological scores					
Short‐term memory domain: DSB	−0.03 ± 0.96	—	0.08 ± 1.08	−0.69	0.49
Short‐term memory domain: DSF	−0.09 ± 1.00	—	0.23 ± 0.97	−2.18	**0.03**
Processing speed domain: TMT‐A	−0.06 ± 1.04	—	0.16 ± 0.87	−1.46	0.15
Processing speed domain: TMT‐B	−0.07 ± 1.04	—	0.19 ± 0.86	−1.81	0.07
Executive functioning domain: SCWT	0.01 ± 0.88	—	−0.02 ± 1.26	0.19	0.85

*Note:* significant (*p* < 0.05) differences are in bold. Where two values of *t* or *χ* ^2^ and *p* are presented, MDD is being compared with both the whole HCC group (left) and the subset with neuropsychology scores (right). Values presented as mean ± SD unless indicated otherwise.

Abbreviations: DSB, digit span backward; DSF, digit span forward; HAMA, Hamilton Anxiety Rating Scale; HAMD, Hamilton Depression Rating Scale; HC, healthy controls; MDD, major depressive disorder; SCWT, stroop color‐word test; SD, standard deviation; TMT‐A, trail making test A; TMT‐B, trail making test B.

^a^ For this MDD versus HC comparison, *p* is from the two‐sample *t*‐test, except.

^b^ *p* from *χ* ^2^ test.

### 3.2 Group Differences in Controllability Metrics

**FIGURE 1:**
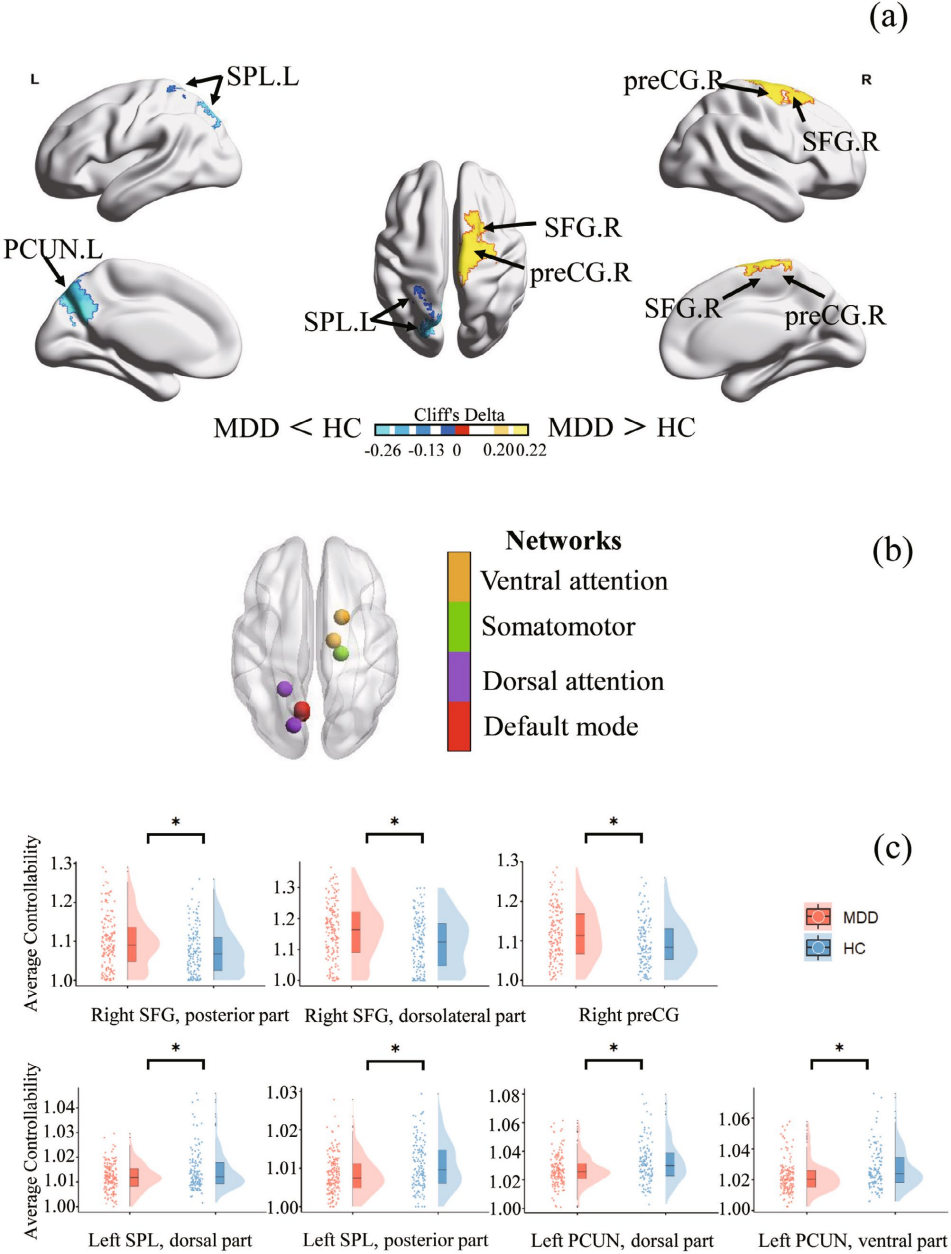
Regional differences in average controllability between MDD patients and HC. (a) Statistical comparison of average controllability distribution between the two groups. The color scale shows the Cliff's *δ* value of the permutation test in both directions of difference. (b) Nodes represent brain regions with altered controllability, based on the Lausanne 2008 template, with the node color indicating the corresponding brain network to which each region belongs. (c) Significant group differences (*p* < 0.05) are marked with an asterisk. Single‐subject controllability values are included as scatterplots. Abbreviations: HC, healthy controls; L, left; MDD, major depressive disorder (first‐episode drug‐naïve); PCUN, precuneus; preCG, precentral gyrus; R, right; SFG, superior frontal gyrus; SPL, superior parietal gyrus.

Compared with HC, MDD patients showed higher AC in the right precentral gyrus (preCG), right dorsolateral SFG, and right posterior SFG, and lower AC in the left dorsal precuneus, left ventral precuneus, left dorsal superior parietal lobule, and left posterior superior parietal lobule. Regions with significant between‐group differences were mainly distributed in the DMN, SMN, and attention network (all FDR‐corrected *p* < 0.05; Figure 1 and Table 2). Although a statistically significant difference was found, the effect sizes were small (Cliff's *δ* < 0.33), suggesting a slight difference between the groups.

**TABLE 2:** Brain nodes showing altered average controllability in first‐episode drug‐naïve MDD patients compared to controls.

Node	Network	MDD	HC	Cliff's *δ*	*p* ^a^
MDD>HC					
Right superior parietal gyrus, posterior part	VAN	1.090 (1.046,1.137)	1.067 (1.023,1.110)	0.214	**0.044**
Right superior parietal gyrus, dorsolateral part	VAN	1.164 (1.090,1.221)	1.124 (1.045,1.186)	0.204	**0.044**
Right precentral gyrus	SMN	1.114 (1.067,1.170)	1.084 (1.050,1.131)	0.217	**0.039**
MDD<HC					
Left superior parietal gyrus, dorsal part	DAN	1.012 (1.008,1.015)	1.014 (1.009,1.018)	−0.130	**0.044**
Left superior parietal gyrus, posterior part	DAN	1.007 (1.004,1.011)	1.011 (1.006,1.015)	−0.232	**0.026**
Left precuneus, dorsal part	DMN	1.026 (1.021,1.031)	1.030 (1.022,1.039)	−0.212	**0.030**
Left precuneus, ventral part	DMN	1.020 (1.015,1.026)	1.024 (1.018,1.034)	−0.255	**0.026**

*Note:* Values presented as median (IQR).

Abbreviations: DAN, dorsal attention network; DMN, default mode network; FDR, false discovery rate; HC, healthy controls; IQR, interquartile range; MDD, major depressive disorder; SD, standard deviation; SMN, somatomotor network; VAN, ventral attention network.

^a^ For this MDD versus HC comparison, *p* is FDR corrected; these are all the significant (*p* < 0.05) differences.

### 3.3 Results of Correlation Analysis

**FIGURE 2:**
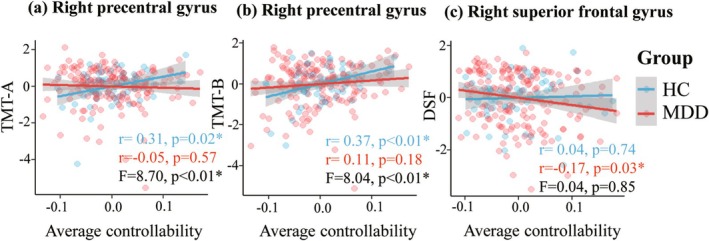
The association between average controllability and cognitive performance in MDD patients and HC. The scatter plots show neuropsychological measures as a function of regional AC in the two groups (see colour key), together with linear regression lines. Data points represent residual values after correcting for age and gender. The AC of the right precentral gyrus was significantly positively associated with processing speed, as measured by (a) TMT‐A and (b) TMT‐B in the HC group; the associations were not significant in the MDD group, and the correlation effects differed significantly between groups (see Section 3 for details) (c) The AC of the posterior part of the right superior frontal gyrus was negatively associated with memory, as measured by the DSF, in the MDD group; although the association was not significant in the HC group, the correlation effects did not differ significantly between groups. *Significant values (*p* < 0.05). DSF, digit span forward; HC, healthy controls; MDD, major depressive disorder; TMT‐A, trail‐making test A; TMT‐B, trail‐making test B.

*Correlations of AC with neuropsychological measures*. In the HC group, AC of the right preCG was significantly positively associated with processing speed as measured by TMT‐A (uncorrected *r* = 0.31, *p* = 0.02) and TMT‐B (uncorrected *r* = 0.37, *p* < 0.01) after controlling for age and sex; in the MDD group, the associations were not significant, and it is therefore not unexpected that the correlation effects differed significantly between groups (*F* = 8.70, *p* < 0.01; *F* = 8.04, *p* < 0.01; respectively) (Figure 2a,b). In the MDD group, AC in the posterior part of the right SFG was negatively associated with memory as measured by DSF (uncorrected *r* = −0.17, *p* = 0.03); in the HC group, the association was not significant, although the correlations did not differ significantly between groups (*F* = 0.04, *p* = 0.85) (Figure 2c). There was no significant correlation between AC alterations and emotional symptoms in the MDD group.

*Correlations between neuropsychological measures*. In the MDD group, HAMD scores were negatively associated with TMT‐B (uncorrected *r* = −0.24, *p* < 0.01) and SCWT (uncorrected *r* = −0.24, *p* < 0.01) scores, while HAMA scores were negatively associated with TMT‐B scores (uncorrected *r* = −0.18, *p* = 0.02) (Table S2): thus, increased severity of depression symptoms was associated with worse processing speed and executive functioning. However, both anxiety and depression symptoms were associated with improvements in memory function: HAMA scores were positively associated with DSB (uncorrected *r* = 0.17, *p* = 0.03) and DSF (uncorrected *r* = 0.23, *p* < 0.01) performance, while HAMD scores were positively associated with DSF (uncorrected *r* = 0.23, p < 0.01) performance (Table S2).

*Demographics correlations*. No significant correlation was found between AC and demographic variables in either the MDD or HC groups.

### 3.4 Results of Moderation and Mediation Analysis

**FIGURE 3:**
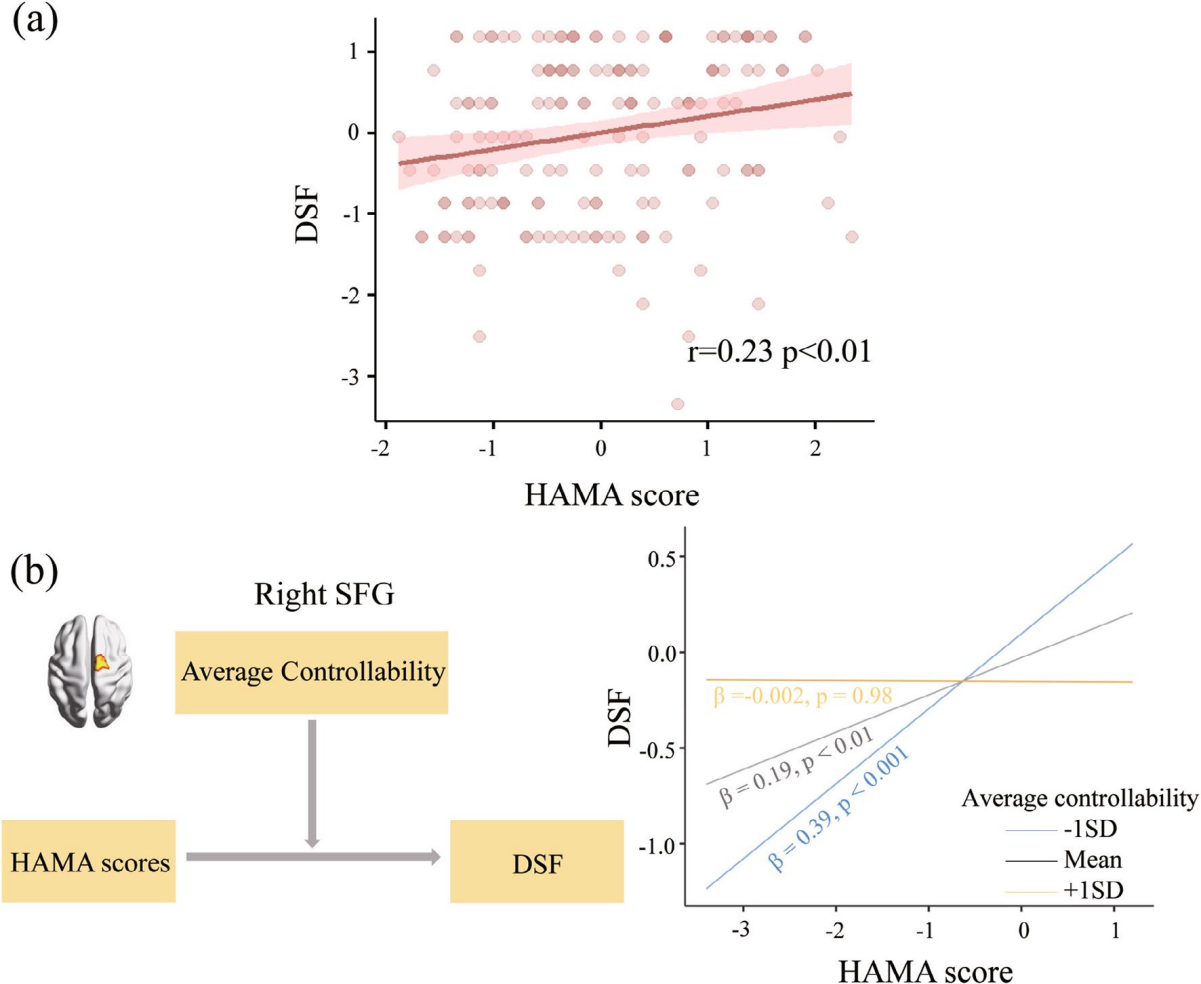
Results of the moderation analysis in the MDD group. In the scatter plot (a), where points represent residual values after correcting for age and gender, higher HAMA scores were associated with improvements in memory, as measured by the DSF. In (b), simple slope analyses revealed that the association between anxiety symptoms and memory performance was stronger when the AC was lower than when it was higher (right‐hand graph). Thus, higher brain average controllability of the posterior part of the right superior frontal gyrus negatively moderated the association between HAMA scores and memory function in the MDD group, as the schematic on the left illustrates. DSF, digit span forward; HAMA, Hamilton Anxiety Rating Scale; MDD, major depressive disorder; SD, standard deviation; SFG, superior frontal gyrus.

In the MDD group only, the AC of the right posterior SFG moderated the association between anxiety symptoms (i.e., HAMA scores) and memory (i.e., DSF scores) (*β* = −0.20, △*R* ^2^ = 0.04, uncorrected *p* = 0.01, see Table S3), but the results did not survive multiple comparison correction. Simple slope analyses revealed that the association between anxiety symptoms and memory performance was stronger when AC was lower (−1SD, *β* = 0.39, *p* < 0.001) than when it was higher (mean, *β* = 0.19, *p* < 0.01; +1SD, *β* = −0.002, *p* = 0.98) (Figure 3). In other words, the AC of the right posterior SFG had a negative moderation effect.

We found no brain regions where AC could mediate the relationship between mood and cognitive symptoms in the MDD group: when AC was included as a mediator in the model, the indirect effects from emotion to controllability or controllability to cognition were not significant (*p* > 0.05).

### 3.5 Relations of Controllability Metrics to White Matter Integrity and Structural Topology

MDD patients showed altered nodal betweenness, nodal degree, nodal efficiency, and FA metrics in regions with altered network controllability, particularly in the precuneus (a major hub of the DMN) (all FDR‐corrected *p* < 0.05; Figure S3 and Table S4). As expected, there was a significant positive correlation between AC and traditional graph theory measurements in both MDD patients and HC (Figure S4 and Table S5). However, the FA measurements correlated weakly with AC in both groups (Table S5).

## 4 Discussion

In the current study, we employed the NCT‐based technique to examine how WM connectivity facilitates the transition between functional brain states. We used AC, as a structural predictor of brain dynamics, to quantify the ability of specific brain regions to drive the system to many easily reachable states. We found higher AC in MDD versus HC in the right preCG and SFG and lower AC in MDD versus HC in the left precuneus and superior parietal lobule; these regions showing MDD versus HC differences are mainly in the DMN, SMN, and attention networks. The patterns of controllability‐cognition correlation differed between MDD patients and HC. Moderation analysis in MDD revealed that the positive association between memory and anxiety symptoms was weaker when the controllability of the right SFG was higher.

### 4.1 Possible Neurobiological Basis of High AC in MDD

Although the biophysical basis of higher AC in MDD is not yet clear, one possibility is that it reflects strong local structural topologies that facilitate the information transitions between brain states that underpin behavioral alterations (Cornblath et al. 2019). Consistent with this, our exploratory analyses revealed that the three nodal centralities (nodal degree, efficiency, and betweenness) of regions showing MDD versus HC differences in AC were positively correlated with AC in both the MDD and HC groups. Specifically, the strong correlation between the nodal degree and AC suggests that densely connected areas are crucial for state transitions (Gu et al. 2017, 2015; Zöller et al. 2021). The associations between AC and nodal efficiency and betweenness may imply that an easily controllable brain system requires a more efficient transmission network (Fang et al. 2021).

### 4.2 Altered AC in MDD

Individuals with MDD showed low AC in the left precuneus, which is a major hub of the DMN. Notably, we observed a consistent reduction in controllability, nodal betweenness, node efficiency, node degree, and FA metrics within the DMN, suggesting that the DMN may represent a critical circuit underlying the pathophysiology of MDD (Kaiser et al. 2015; Scalabrini et al. 2020). The DMN is considered to be in a pluripotent ‘ground state’ and has the potential to direct brain function to various task‐specific states (Lin et al. 2017). Our findings suggest an impaired ability of the DMN to switch between task‐relevant brain systems, perhaps related to known lower connectivity between the DMN and other networks in MDD (Wang, Wang, et al. 2020). The diminished ability to shift in and out of the DMN dominance in activity may impair the functional balance between the DMN and other task networks, which is essential for effectively allocating cognitive resources in response to environmental demands (Tang et al. 2023; Whitfield‐Gabrieli and Ford 2012). In view of earlier reports of lower nodal degree and efficiency of the DMN in MDD versus HC (Chen et al. 2017; Yang et al. 2021), it is not surprising that we found a decreasing trend in controllability in MDD patients, considering the positive association between controllability and nodal centrality metrics (Figure S3 and Table S5).

Controllability of the right preCG, a region of the SMN, was significantly higher in MDD patients. Depression‐related gray matter volume atrophy (Yu et al. 2023) and functional abnormalities during specific tasks (Wang, Du, et al. 2015) and during rest (Javaheripour et al. 2021; Yao et al. 2019) in this region are regularly reported in MDD. Disruptions in functional connectivity (FC) from SMN to DMN, VAN, and VN have been shown to associate with psychomotor retardation in MDD (Northoff et al. 2021). Patients with depression exhibit reduced movement and activity, which, at the neuronal level, may be closely related to dysfunction in the SMN (Martino et al. 2016). Higher AC in the right preCG in MDD may suggest an increased ability to facilitate transitions away from brain states associated with motor activity. Previous studies have shown that increased local functional connectivity within the SMN after treatment in MDD patients was associated with improvement in clinical symptoms (Wang et al. 2018). Others reported hyperactivity in the preCG during motor control‐related tasks in MDD (Fitzgerald et al. 2008). In fact, our univariate correlation analysis also found that higher AC of this region was associated with better processing speed performance. Thus, we speculated that higher AC in the right preCG observed in our study may reflect the compensatory efforts to adjust for movement executions in MDD patients.

MDD patients versus HC showed lower AC in the left superior parietal lobule but higher AC in the right SFG. The left superior parietal lobule is a major hub of the DAN engaged in goal‐oriented activities (Corbetta et al. 2008); it is plausible that with lower AC in the DAN, the control of attention maintenance is impaired. Conversely, the right posterior SFG belongs to the VAN, which is important in the stimulus‐driven reorientation of attention (Corbetta et al. 2008), and here higher AC may facilitate rapid adaptation to the environment, a self‐protection strategy in depressed patients (Farrant and Uddin 2015), but perhaps at some cost to activities that demand attention control (Keller et al. 2019).

The dysfunction of VAN–DAN interaction indicates the disturbance of brain attention resource mobilization during externally directed tasks (Corbetta et al. 2008). Increased excitatory activity within the DMN and reduced inhibitory transmission from the DMN to the VAN may reflect the inability to effectively switch internal and external patterns of attention in patients with MDD (Wang et al. 2022, 2024). Other research linked the incapacity to distract attention from negative stimuli to the lower FC between SMN and VAN (Hilland et al. 2018). Taken together, abnormal interactions among the DMN, SMN, and attention network could be related to disruptions in the balance between internal states and external awareness, which may reflect biases toward ruminative thoughts (Kaiser et al. 2015).

Although we identified MDD versus HC differences in AC across several brain regions, the effect size was small, which may reflect interindividual heterogeneity and the subtle nature of structural alterations in MDD (Winter et al. 2022; Yarkoni 2009). However, these subtle brain alterations, when correlated with behavioral features, may indicate a broader network‐level dysfunction, potentially representing the cumulative result of multiple interacting factors (Genon et al. 2022).

### 4.3 Cognitive Defects and Altered AC in MDD

Our MDD patients (mean age 31 years) demonstrated mainly impaired memory, rather than defects in processing speed and executive functioning. This may reflect the age‐related cognitive deficit phenomenon in MDD: worse executive function and processing speed in late‐life depression versus memory deficits in young depressed patients (Castaneda et al. 2008; Herrmann et al. 2007; Marazziti et al. 2010).

The patterns of controllability‐cognition correlation differed in MDD versus HC: in the HC group, there were significant positive correlations between cognitive function and AC in the right preCG, with a weak trend in the same direction in the right SFG; however, these correlations were reduced or even reversed in the MDD group (Figure 2). Of note, these correlation analyses results did not remain significant after correcting for multiple comparisons with FDR. Processing speed and short‐term memory rely on the functioning of large‐scale, long‐distance neural networks that are supported by myelinated fibers (Bartzokis et al. 2010; Felts et al. 1997; Kochunov et al. 2017). The importance of WM microstructure in supporting processing speed and memory measures has been observed in both healthy individuals and patients with MDD (Glahn et al. 2013; Liang et al. 2019; Penke et al. 2010; Smith et al. 2011; Yamada et al. 2015). Given the MDD versus HC differences in brain controllability based on WM connectivity, these differences in the association patterns may reflect the impact of WM pathway abnormalities on specific cognitive domains in MDD patients, particularly in processing speed and short‐term memory functions.

In addition, severe depressive and anxiety symptoms were related to poorer executive functioning and processing speed, which were consistent with previous studies (Basso et al. 2007; Matcham et al. 2023; Withall et al. 2009). Potential mechanisms related to these findings may involve dysregulation of cortisol levels resulting in neurological impairments (Hu et al. 2023). However, we obtained the opposite results regarding memory function, which seem to contradict prior findings (McClintock et al. 2010; Naismith et al. 2003). Although we found that the AC in the right SFG can moderate the relationship between memory and anxiety symptoms, a positive correlation between memory and anxiety symptoms persisted even at lower AC levels (Figure 3b). One possible explanation is that additional moderating psychosocial factors (such as educational attainment, socio‐economic status, and pressure) may alter the initial negative association between clinical symptoms and memory in MDD patients (O'Shea et al. 2015; Opdebeeck et al. 2018). We may also speculate that individuals with severe depressive and anxiety symptoms tend to be excessively critical of their own performance, striving to perform better in order to avoid leaving a negative impression on the examiner (Balash et al. 2013; Beblo et al. 2020). Why this phenomenon occurs only in the memory domain will be an attractive topic for future research. All associations with cognition should be treated with caution as they did not survive multiple comparison correction.

In the formal moderation analysis, we found that the effect of anxiety symptoms on memory function was weaker in the presence of relatively higher AC in the right SFG, but the result did not survive multiple comparison correction. Supporting this, the correlational analysis revealed that a relatively higher AC in this region was associated with worse memory performance in MDD patients. Given the critical role noted above of right SFG activity in stimulus‐driven reorientation of attention (Corbetta et al. 2008), the high AC of this region may facilitate frequent alterations in attention focus as well as behavioral distractions, which may impair storage and retrieval processes of memory (Wang, Zhou, et al. 2020). Overall, our study highlights the potential role of measures of controllability in the interaction between anxiety and memory in MDD.

### 4.4 Limitations of This Study

First, the use of non‐isovoxels DTI parameters in our study may introduce anisotropy distortions and reduce spatial resolution (Kim et al. 2006). Future studies could benefit from exploring the impact of using alternative DTI parameters on the results. Second, the NCT approach is built on a linear model of neuronal dynamics. Although this assumption clearly oversimplifies the brain dynamic system, it satisfactorily explains the sort of brain activity variations captured by fMRI data (Cornblath et al. 2020). As methods evolve, future studies may consider nonlinear neural modelling and, even more challenging, integration with real fMRI data. Third, although we made optimal use of the available data, the imbalance of cognitive data between MDD patients and HC might have limited the statistical power and generalizability of the current study. Fourth, caution is warranted when interpreting group differences with small effect sizes (Cliff's *δ* < 0.33), while these modest regional‐level alterations likely reflect subtle but significant components of a broader network‐level dysfunction underlying MDD (Genon et al. 2022). Fifth, the results of the univariate correlation and moderation analyses should be treated with caution as they did not survive multiple comparison correction. Sixth, we chose three comprehensive topological indices to explore the potential pathophysiological implications of alterations in controllability; additional graph theory indices could provide complementary information. Finally, the cross‐sectional design precluded us from ascertaining causality: whether persistent cognitive dysfunction precedes emotional symptoms, or whether the severity of emotional symptoms contributes to cognitive deficits, or (perhaps more likely) the relationship is bidirectional (Gonda et al. 2015).

## 5 Conclusions

Using the NCT approach, we demonstrated altered brain AC in the DMN, SMN, and attention network in first‐episode drug‐naïve MDD patients, providing a novel understanding of the neural substrate underlying cognitive control. Patterns of controllability cognition correlation differed between MDD and HC, which may reflect the impact of WM pathway damage on typical cognition associations in MDD patients. Our study highlights the potential role of measures of controllability in the interaction between anxiety and memory in MDD.

## Author Contributions

Aoxiang Zhang, Qian Zhang, and Youjin Zhao designed and organized the study. Aoxiang Zhang, Qian Zhang, Ziyuan Zhao, Qian Li, Yongbo Hu, and Youjin Zhao collected the data. Aoxiang Zhang, Qian Zhang, Xiaoqi Huang, and Weihong Kuang analyzed the data. Aoxiang Zhang and Qian Zhang wrote and revised the manuscript, which was also critically revised by Graham Kemp and Fei Li. Youjin Zhao and Qiyong Gong supervised the project and secured funding support.

## Conflicts of Interest

The authors declare no conflicts of interest.

## Supporting information


**Data S1.** Supporting Information.

## Data Availability

All the data are in principle available for research purposes.
