# Supplementary material for: The Neural Association Between Symptom and Cognition in Major Depressive Disorder: A Network Control Theory Study
Source: Hum Brain Mapp. 2025 Mar 20;46(5):e70198. doi: 10.1002/hbm.70198 (PMC11923719; doi:10.1002/hbm.70198)
Supplement: Supplementary file 1 — Data S1. Supporting Information. [file HBM-46-e70198-s001.docx]

**SUPPLEMENT**

**The neural association between symptom and cognition in major depressive disorder：A network control theory study**

**Contents**

**Supplementary Methods**

**FA metrics and structural topological calculations**

*Voxel-based analysis*

*Graph theory analysis*

*Correlation analysis*

*Comparison of controllability metrics with traditional graph-theoretic and white matter integrity metrics*

**Table S1**. The 234 brain regions in the Lausanne 2008 Atlas

**Figure S1**. Area under the curve (AUC) calculation for network metrics.

**Supplementary Results**

**Figure S2** Histogram of frequency distribution for average controllability in brain regions with MDD *vs* HC differences.

**Figure S3** Controllability versus graph measures.

**Figure S4** Associations in MDD patients between AC and regional nodal centralities metrics.

**Table S2** Correlations between symptom and cognitive scores in MDD patients.

**Table S3** AC of right superior frontal gyrus moderates the association between memory and anxiety symptoms in MDD patients.

**Table S4** Comparison of nodal centralities and FA metrics in MDD *vs* HC in brain regions which show altered AC in MDD patients

**Table S5** Associations in MDD and HC groups of AC with nodal centralities and FA measures.

# Supplementary Methods

## FA metrics and structural topological calculations

### Voxel-based analysis

We used PANDA to extract and calculate the whole-brain diffusion metric FA. The regional FA value of each region was calculated by averaging the FA values of all voxels within each region of the Lausanne 2008 atlas (Cammoun et al., 2012; Hagmann et al., 2008).

### Graph theory analysis

Structural topological metrics were calculated using GRETNA (www.nitrc.org/projects/gretna/) software (Wang et al., 2015). First, we obtained an anatomical 234×234 matrix for each participant. We then selected a range of sparsity thresholds for each WM connectivity matrix based on the following two criteria: (1) the average degree (the number of connections associated with a node) of all nodes for each threshold network exceeded 2 × log (234); and (2) the small-world parameter of the threshold network for each subject exceeded 1.0 (Watts & Strogatz, 1998). The thresholds were determined based on these criteria with a range of 0.02 to 0.5 and an interval of 0.01. We calculated the nodal metrics of particular regions at each level of sparsity. The area under the curve (AUC) was calculated for each nodal metric to obtain a cost-threshold-invariant measure of the topological properties of brain networks (Figure S1) (Achard & Bullmore, 2007; Zhang et al., 2011). The three regional nodal centrality metrics we used evaluate the role of a node in a network from different perspectives: the nodal degree measures the total number of connections a node forms with other nodes, which reflects its ability to exchange information in a functional network (Rubinov & Sporns, 2010); nodal betweenness denotes the number of shortest paths through a node and reflects its impact on the flow of information between other nodes (Rubinov & Sporns, 2010); and nodal efficiency refers to the capacity of a node to exchange information with other nodes in a network (Achard & Bullmore, 2007).

### Correlation analysis

For regions showing group differences in AC, we used Spearman's rank correlation analysis, with age and sex as covariates, to assess the associations in the MDD group between AC and emotional symptoms (HAMD and HAMA scores) and cognitive functioning (DSB, DSF, TMT-A, TMT-B, and SCWT scores), and the associations in the HC group between AC values and cognitive variables (DSB, DSF, TMT-A, TMT-B, and SCWT scores). Similarly, we assessed the association between AC and demographic characteristics: age and illness in the MDD group, and age in the HC group.

### Comparison of controllability metrics with traditional graph-theoretic and white matter integrity metrics

First, we compared regional nodal centralities (nodal degree, nodal efficiency, and nodal betweenness) and WM integrity metric (FA) in the two groups using permutation tests, with age and sex as covariates. The false discovery rate (FDR) correction method for multiple comparisons was performed, accounting for a total of 234 statistical corrections. Second, in regions showing significant AC differences between MDD patients and HC, partial Spearman's rank correlation analyses were used to examine the associations of AC with nodal centrality metrics and FA in the MDD and HC groups; age and sex were treated as covariates, and findings were corrected with FDR (14 corrections) to preserve a p<0.05 threshold.

**Table S1.** The 234 brain regions in the Lausanne 2008 Atlas

| **LN** | **Lausanne (Right)** | **Yeo** | **LN** | **Lausanne (Left)** | **Yeo** |
| --- | --- | --- | --- | --- | --- |
| 1 | lateralorbitofrontal_1 | Limbic | 118 | lateralorbitofrontal_3 | Frontoparietal Control |
| 2 | lateralorbitofrontal_2 | Default Mode | 119 | lateralorbitofrontal_4 | Limbic |
| 3 | lateralorbitofrontal_3 | Frontoparietal Control | 120 | parsorbitalis_1 | Default Mode |
| 4 | lateralorbitofrontal_4 | Limbic | 121 | frontalpole_1 | Limbic |
| 5 | parsorbitalis_1 | Default Mode | 122 | medialorbitofrontal_1 | Default Mode |
| 6 | frontalpole_1 | Limbic | 123 | medialorbitofrontal_2 | Limbic |
| 7 | medialorbitofrontal_1 | Default Mode | 124 | parstriangularis_1 | Default Mode |
| 8 | medialorbitofrontal_2 | Limbic | 125 | parsopercularis_1 | Frontoparietal Control |
| 9 | medialorbitofrontal_3 | Limbic | 126 | parsopercularis_2 | Default Mode |
| 10 | parstriangularis_1 | Ventral Attention | 127 | rostralmiddlefrontal_1 | Frontoparietal Control |
| 11 | parstriangularis_2 | Frontoparietal Control | 128 | rostralmiddlefrontal_2 | Ventral Attention |
| 12 | parsopercularis_1 | Frontoparietal Control | 129 | rostralmiddlefrontal_3 | Frontoparietal Control |
| 13 | parsopercularis_2 | Frontoparietal Control | 130 | rostralmiddlefrontal_4 | Default Mode |
| 14 | rostralmiddlefrontal_1 | Frontoparietal Control | 131 | rostralmiddlefrontal_5 | Frontoparietal Control |
| 15 | rostralmiddlefrontal_2 | Frontoparietal Control | 132 | rostralmiddlefrontal_6 | Default Mode |
| 16 | rostralmiddlefrontal_3 | Frontoparietal Control | 133 | superiorfrontal_1 | Default Mode |
| 17 | rostralmiddlefrontal_4 | Frontoparietal Control | 134 | superiorfrontal_2 | Default Mode |
| 18 | rostralmiddlefrontal_5 | Default Mode | 135 | superiorfrontal_3 | Default Mode |
| 19 | rostralmiddlefrontal_6 | Frontoparietal Control | 136 | superiorfrontal_4 | Default Mode |
| 20 | superiorfrontal_1 | Default Mode | 137 | superiorfrontal_5 | Default Mode |
| 21 | superiorfrontal_2 | Default Mode | 138 | superiorfrontal_6 | Default Mode |
| 22 | superiorfrontal_3 | Default Mode | 139 | superiorfrontal_7 | Ventral Attention |
| 23 | superiorfrontal_4 | Default Mode | 140 | superiorfrontal_8 | Somatomotor |
| 24 | superiorfrontal_5 | Ventral Attention | 141 | superiorfrontal_9 | Dorsal Attention |
| 25 | superiorfrontal_6 | Ventral Attention | 142 | caudalmiddlefrontal_1 | Default Mode |
| 26 | superiorfrontal_7 | Ventral Attention | 143 | caudalmiddlefrontal_2 | Frontoparietal Control |
| 27 | superiorfrontal_8 | Frontoparietal Control | 144 | caudalmiddlefrontal_3 | Dorsal Attention |
| 28 | caudalmiddlefrontal_1 | Frontoparietal Control | 145 | precentral_1 | Somatomotor |
| 29 | caudalmiddlefrontal_2 | Frontoparietal Control | 146 | precentral_2 | Somatomotor |
| 30 | caudalmiddlefrontal_3 | Frontoparietal Control | 147 | precentral_3 | Somatomotor |
| 31 | precentral_1 | Ventral Attention | 148 | precentral_4 | Somatomotor |
| 32 | precentral_2 | Dorsal Attention | 149 | precentral_5 | Dorsal Attention |
| 33 | precentral_3 | Somatomotor | 150 | precentral_6 | Somatomotor |
| 34 | precentral_4 | Somatomotor | 151 | precentral_7 | Dorsal Attention |
| 35 | precentral_5 | Somatomotor | 152 | precentral_8 | Ventral Attention |
| 36 | precentral_6 | Somatomotor | 153 | paracentral_1 | Somatomotor |
| 37 | paracentral_1 | Somatomotor | 154 | paracentral_2 | Somatomotor |
| 38 | paracentral_2 | Somatomotor | 155 | rostralanteriorcingulate_1 | Default Mode |
| 39 | paracentral_3 | Ventral Attention | 156 | caudalanteriorcingulate_1 | Ventral Attention |
| 40 | rostralanteriorcingulate_1 | Default Mode | 157 | posteriorcingulate_1 | Frontoparietal Control |
| 41 | caudalanteriorcingulate_1 | Ventral Attention | 158 | posteriorcingulate_2 | Ventral Attention |
| 42 | posteriorcingulate_1 | Ventral Attention | 159 | isthmuscingulate_1 | Default Mode |
| 43 | posteriorcingulate_2 | Frontoparietal Control | 160 | postcentral_1 | Somatomotor |
| 44 | isthmuscingulate_1 | Default Mode | 161 | postcentral_2 | Somatomotor |
| 45 | postcentral_1 | Somatomotor | 162 | postcentral_3 | Somatomotor |
| 46 | postcentral_2 | Somatomotor | 163 | postcentral_4 | Somatomotor |
| 47 | postcentral_3 | Somatomotor | 164 | postcentral_5 | Somatomotor |
| 48 | postcentral_4 | Somatomotor | 165 | postcentral_6 | Somatomotor |
| 49 | postcentral_5 | Somatomotor | 166 | postcentral_7 | Somatomotor |
| 50 | supramarginal_1 | Dorsal Attention | 167 | supramarginal_1 | Somatomotor |
| 51 | supramarginal_2 | Frontoparietal Control | 168 | supramarginal_2 | Ventral Attention |
| 52 | supramarginal_3 | Ventral Attention | 169 | supramarginal_3 | Ventral Attention |
| 53 | supramarginal_4 | Somatomotor | 170 | supramarginal_4 | Default Mode |
| 54 | superiorparietal_1 | Dorsal Attention | 171 | supramarginal_5 | Dorsal Attention |
| 55 | superiorparietal_2 | Dorsal Attention | 172 | superiorparietal_1 | Dorsal Attention |
| 56 | superiorparietal_3 | Dorsal Attention | 173 | superiorparietal_2 | Dorsal Attention |
| 57 | superiorparietal_4 | Dorsal Attention | 174 | superiorparietal_3 | Dorsal Attention |
| 58 | superiorparietal_5 | Dorsal Attention | 175 | superiorparietal_4 | Dorsal Attention |
| 59 | superiorparietal_6 | Visual | 176 | superiorparietal_5 | Dorsal Attention |
| 60 | superiorparietal_7 | Visual | 177 | superiorparietal_6 | Visual |
| 61 | inferiorparietal_1 | Frontoparietal Control | 178 | superiorparietal_7 | Visual |
| 62 | inferiorparietal_2 | Default Mode | 179 | inferiorparietal_1 | Visual |
| 63 | inferiorparietal_3 | Frontoparietal Control | 180 | inferiorparietal_2 | Default Mode |
| 64 | inferiorparietal_4 | Default Mode | 181 | inferiorparietal_3 | Default Mode |
| 65 | inferiorparietal_5 | Default Mode | 182 | inferiorparietal_4 | Default Mode |
| 66 | inferiorparietal_6 | Visual | 183 | inferiorparietal_5 | Frontoparietal Control |
| 67 | precuneus_1 | Visual | 184 | precuneus_1 | Dorsal Attention |
| 68 | precuneus_2 | Default Mode | 185 | precuneus_2 | Default Mode |
| 69 | precuneus_3 | Default Mode | 186 | precuneus_3 | Default Mode |
| 70 | precuneus_4 | Default Mode | 187 | precuneus_4 | Default Mode |
| 71 | precuneus_5 | Dorsal Attention | 188 | precuneus_5 | Default Mode |
| 72 | cuneus_1 | Visual | 189 | cuneus_1 | Visual |
| 73 | cuneus_2 | Visual | 190 | pericalcarine_1 | Visual |
| 74 | pericalcarine_1 | Visual | 191 | lateraloccipital_1 | Visual |
| 75 | pericalcarine_2 | Visual | 192 | lateraloccipital_2 | Visual |
| 76 | lateraloccipital_1 | Visual | 193 | lateraloccipital_3 | Visual |
| 77 | lateraloccipital_2 | Visual | 194 | lateraloccipital_4 | Visual |
| 78 | lateraloccipital_3 | Visual | 195 | lateraloccipital_5 | Visual |
| 79 | lateraloccipital_4 | Visual | 196 | lingual_1 | Visual |
| 80 | lateraloccipital_5 | Visual | 197 | lingual_2 | Visual |
| 81 | lingual_1 | Visual | 198 | lingual_3 | Visual |
| 82 | lingual_2 | Visual | 199 | lingual_4 | Visual |
| 83 | lingual_3 | Visual | 200 | fusiform_1 | Visual |
| 84 | fusiform_1 | Visual | 201 | fusiform_2 | Dorsal Attention |
| 85 | fusiform_2 | Visual | 202 | fusiform_3 | Visual |
| 86 | fusiform_3 | Visual | 203 | fusiform_4 | Limbic |
| 87 | fusiform_4 | Limbic | 204 | parahippocampal_1 | Default Mode |
| 88 | parahippocampal_1 | Visual | 205 | entorhinal_1 | Limbic |
| 89 | entorhinal_1 | Limbic | 206 | temporalpole_1 | Limbic |
| 90 | temporalpole_1 | Limbic | 207 | inferiortemporal_1 | Limbic |
| 91 | inferiortemporal_1 | Limbic | 208 | inferiortemporal_2 | Limbic |
| 92 | inferiortemporal_2 | Limbic | 209 | inferiortemporal_3 | Dorsal Attention |
| 93 | inferiortemporal_3 | Frontoparietal Control | 210 | inferiortemporal_4 | Dorsal Attention |
| 94 | inferiortemporal_4 | Dorsal Attention | 211 | middletemporal_1 | Ventral Attention |
| 95 | middletemporal_1 | Frontoparietal Control | 212 | middletemporal_2 | Default Mode |
| 96 | middletemporal_2 | Default Mode | 213 | middletemporal_3 | Default Mode |
| 97 | middletemporal_3 | Default Mode | 214 | middletemporal_4 | Default Mode |
| 98 | middletemporal_4 | Default Mode | 215 | bankssts_1 | Default Mode |
| 99 | bankssts_1 | Ventral Attention | 216 | bankssts_2 | Default Mode |
| 100 | superiortemporal_1 | Somatomotor | 217 | superiortemporal_1 | Somatomotor |
| 101 | superiortemporal_2 | Somatomotor | 218 | superiortemporal_2 | Somatomotor |
| 102 | superiortemporal_3 | Somatomotor | 219 | superiortemporal_3 | Default Mode |
| 103 | superiortemporal_4 | Ventral Attention | 220 | superiortemporal_4 | Somatomotor |
| 104 | superiortemporal_5 | Default Mode | 221 | superiortemporal_5 | Limbic |
| 105 | transversetemporal_1 | Somatomotor | 222 | transversetemporal_1 | Somatomotor |
| 106 | insula_1 | Somatomotor | 223 | insula_1 | Somatomotor |
| 107 | insula_2 | Ventral Attention | 224 | insula_2 | Ventral Attention |
| 108 | insula_3 | Ventral Attention | 225 | insula_3 | Ventral Attention |
| 109 | thalamus proper | Subcortical | 226 | insula_4 | Ventral Attention |
| 110 | caudate | Subcortical | 227 | Thalamus proper | Subcortical |
| 111 | putamen | Subcortical | 228 | caudate | Subcortical |
| 112 | pallidum | Subcortical | 229 | putamen | Subcortical |
| 113 | accumbens | Subcortical | 230 | pallidum | Subcortical |
| 114 | hippocampus | Subcortical | 231 | accumbens | Subcortical |
| 115 | amygdala | Subcortical | 232 | hippocampus | Subcortical |
| 116 | lateralorbitofrontal_1 | Limbic | 233 | amygdala | Subcortical |
| 117 | lateralorbitofrontal_2 | Default Mode | 234 | brainstem | Subcortical |

**Fig. S1. Area under the curve (AUC) calculation for network metrics**

The AUC (shaded area) for a network metric Y over the sparsity threshold range S_1_ to S_n_ with interval ΔS, is computed as $Y^{\mathrm{AUC}}=\sum_{k=1}^{n-1} [Y\left( S_{k} \right)+Y\left( S_{k+1} \right)]\times\Delta S/2 ADDIN EN.CITE ADDIN EN.CITE.DATA (Zhang et al., 2011)$. In this study S_1_= 0.02, S_n_ = 0.5 and ΔS = 0.01.


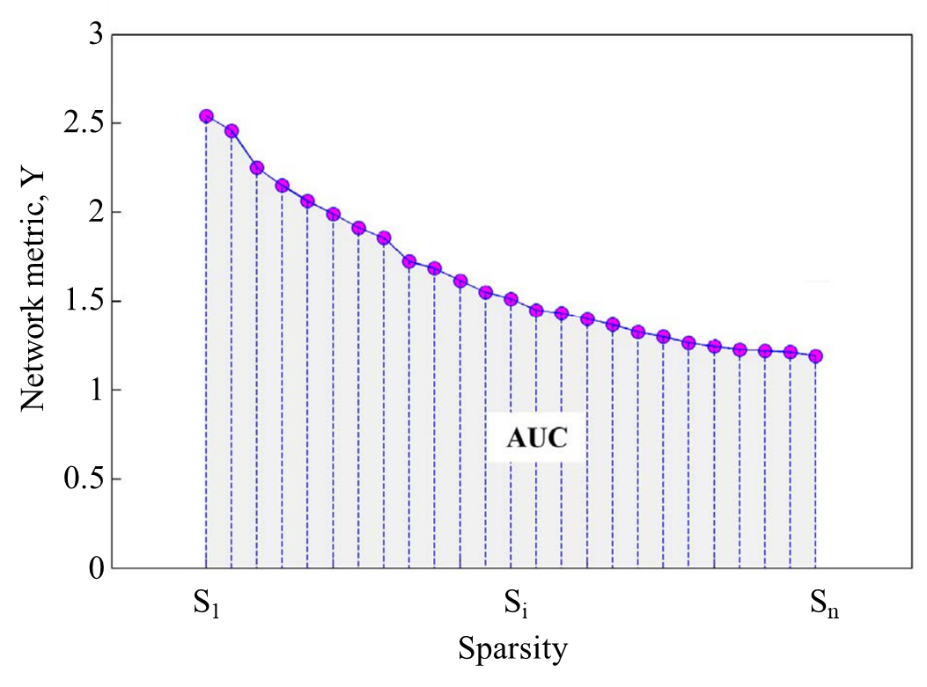


# Supplementary Results

**Fig. S2**. **Histogram of** **frequency distribution for average controllability in brain regions with MDD *vs* HC differences.** Abbreviations: HC, healthy controls; MDD, major depressive disorder. PCUN, precuneus; preCG, precentral gyrus; SFG, superior frontal gyrus; SPL, superior parietal gyrus.


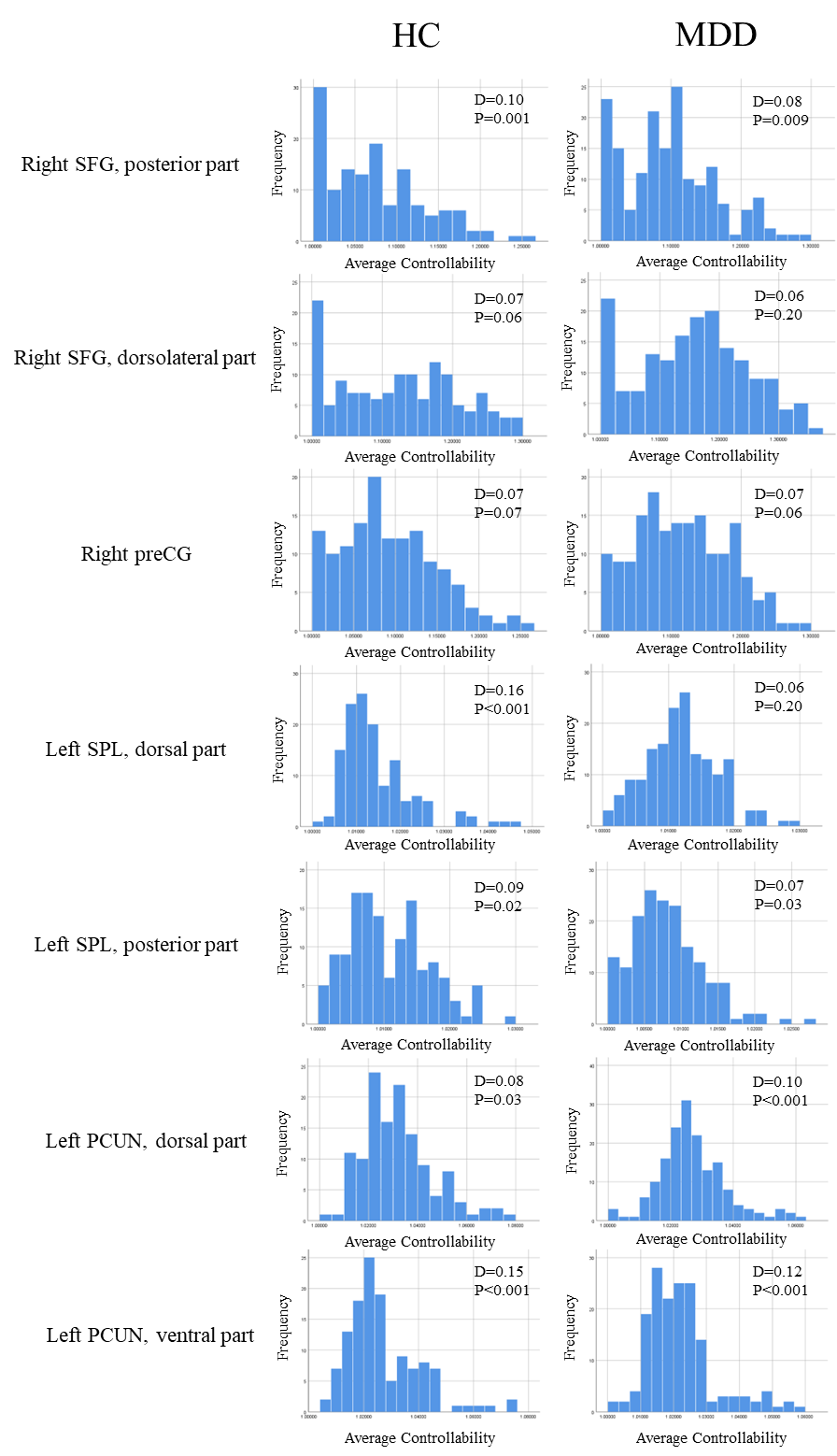


**Fig. S3. Controllability versus graph measures.** Nodes represent brain regions with altered NB, NE, ND, and AC in MDD patients compared to HC at whole-brain level, with the node color indicating the corresponding brain network to which each region belongs. Abbreviations: DAN, dorsal attention network; DMN, default mode network; FPN, frontoparietal control network; HC, healthy controls; L, left; MDD, major depressive disorder; NB, nodal betweenness, ND; nodal degree; NE nodal efficiency; PCC, posterior cingulate cortex; PCUN, precuneus; preCG, precentral gyrus; R, right; SFG, superior frontal gyrus; SMN, somatomotor network; SPL, superior parietal gyrus; VAN, ventral attention network.


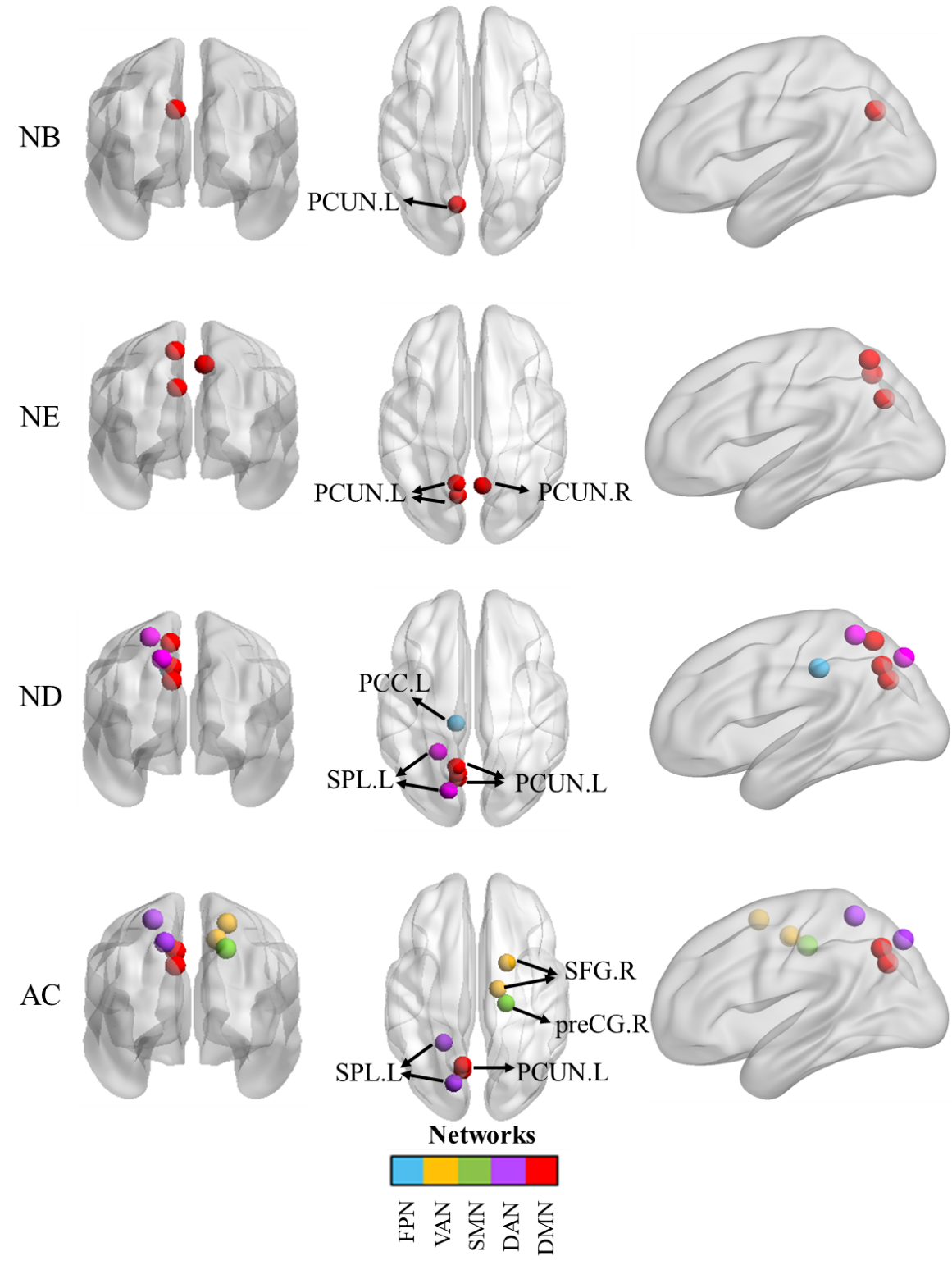


**Fig. S4**. **Associations in MDD patients between AC and regional nodal metrics in three brain regions.** The AC of (a) right precentral gyrus, (b) left superior parietal gyrus and (c) left precuneus (c) was positively associated with the nodal degree (left) and nodal efficiency (right) in MDD patients. Abbreviations: AC, average controllability; MDD, major depressive disorder.


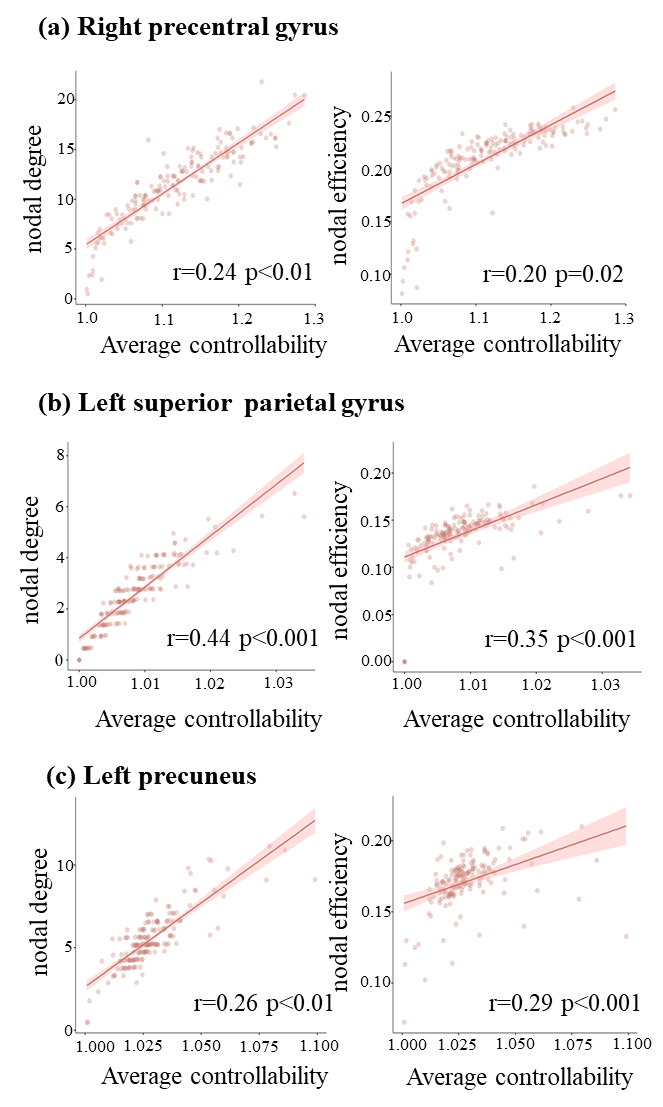


**Table S2.** Correlations in MDD patients between symptom and cognitive scores

|  | HAMA | |  | HAMD | |
| --- | --- | --- | --- | --- | --- |
|  | r | p |  | r | p |
| DSB | 0.17 | **0.03** |  | 0.06 | 0.46 |
| DSF | 0.23 | **<0.01** |  | 0.22 | **<0.01** |
| TMT-A | -0.08 | 0.29 |  | -0.16 | **0.04** |
| TMT-B | -0.18 | **0.02** |  | -0.24 | **<0.01** |
| SCWT | -0.05 | 0.53 |  | -0.24 | **<0.01** |

p<0.05 in bold. Abbreviations: DSB, Digit Span Backward; DSF, Digit Span Forward; HAMA, Hamilton Anxiety Rating Scale; HAMD, Hamilton Depression Rating Scale; MDD, major depressive disorder; SCWT, Stroop Color-Word Test; TMT-A, Trail Making Test A; TMT-B, Trail Making Test B.

**Table S3.** AC of right superior frontal gyrus moderates the association between memory and anxiety symptoms in MDD patients

| Dependent  variable | Independent  variable | △R^2^ | β | SE. | t | p | 95% CI |
| --- | --- | --- | --- | --- | --- | --- | --- |
| DSF scores |  | **0.04** |  |  |  |  |  |
|  | HAMA |  | 0.19 | 0.08 | 2.57 | **<0.01** | [0.05, 0.34] |
|  | AC |  | -0.12 | 0.07 | -1.64 | 0.10 | [-0.27, 0.03] |
|  | HAMA*AC |  | -0.20 | 0.08 | -2.60 | **<0.01** | [-0.35, -0.05] |

p<0.05 in bold. Abbreviations: AC, average controllability; CI, confidence interval; DSF, Digit Span Forward; HAMA, Hamilton Anxiety Rating Scale; HAMD, Hamilton Depression Rating Scale; MDD, major depressive disorder; SE, standard error.

**Table S4.** Comparison of nodal centralities and FA metrics in MDD *vs* HC in brain regions which show altered AC in MDD patients

| Region | Metric | MDD | HC | Delta | p* |
| --- | --- | --- | --- | --- | --- |
|  | BC | 91.419 (31.868, 187.329) | 49.021 (8.658, 159.984) | 0.172 | 0.172 |
| Right SFG, | DC | 7.613 (5.227, 9.516) | 6.605 (3.983, 8.580) | 0.186 | 0.108 |
| posterior part | NE | 0.207 (0.191, 0.217) | 0.202 (0.179, 0.213) | 0.154 | 0.360 |
|  | FA | 0.144 (0.139, 0.151) | 0.165 (0.158, 0.171) | -0.132 | **0.024** |
|  | BC | 272.906 (91.551, 592.949) | 199.654 (60.957, 417.207) | 0.162 | 0.112 |
| Right SFG, | DC | 11.425 (8.515, 13.991) | 10.485 (6.603, 13.265) | 0.155 | 0.147 |
| dorsolateral part | NE | 0.222 (0.207, 0.234) | 0.217 (0.192, 0.231) | 0.137 | 0.360 |
|  | FA | 0.235 (0.229, 0.243) | 0.147 (0.139, 0.154) | -0.020 | 0.483 |
| Right preCG | BC | 445.176 (231.050, 817.619) | 345.936 (187.972, 614.014) | 0.179 | 0.131 |
|  | DC | 11.680 (8.994, 14.229) | 10.405 (8.240, 12.753) | 0.155 | 0.142 |
|  | NE | 0.219 (0.201, 0.232) | 0.212 (0.195, 0.227) | 0.126 | 0.393 |
|  | FA | 0.236 (0.230, 0.243) | 0.237 (0.231, 0.244) | -0.070 | 0.098 |
|  | BC | 9.838 (2.824, 33.702) | 14.924 (5.902, 39.244) | -0.166 | 0.376 |
| Left SPL, | DC | 3.320 (2.800, 4.233) | 3.680 (2.813, 4.643) | -0.184 | **0.008** |
| dorsal part | NE | 0.147 (0.136, 0.155) | 0.152 (0.143, 0.161) | -0.187 | 0.067 |
|  | FA | 0.170 (0.164, 0.178) | 0.172 (0.166, 0.179) | -0.115 | 0.080 |
|  | BC | 4.906 (0.532, 4.906) | 7.147 (1.456, 22.256) | -0.127 | 0.351 |
| Left SPL, | DC | 2.700 (1.806, 3.354) | 2.845 (2.255, 3.760) | -0.216 | **0.019** |
| posterior part | NE | 0.139 (0.129, 0.148) | 0.147 (0.133, 0.155) | -0.250 | 0.360 |
|  | FA | 0.114 (0.106, 0.119) | 0.110 (0.104, 0.116) | 0.205 | 0.119 |
|  | BC | 65.208 (29.830, 116.315) | 88.632 (38.978, 143.246) | -0.166 | 0.131 |
| Left PCUN, | DC | 5.210 (4.289, 6.268) | 6.140 (4.718, 7.973) | -0.259 | **0.008** |
| dorsal part | NE | 0.173 (0.164, 0.181) | 0.180 (0.168, 0.191) | -0.234 | 0.109 |
|  | FA | 0.205 (0.199, 0.212) | 0.211 (0.202, 0.219) | -0.213 | **0.002** |
|  | BC | 56.831 (21.713, 116.437) | 95.484 (36.077, 161.356) | -0.238 | **0.047** |
| Left PCUN, | DC | 4.688 (3.765, 5.599) | 5.535 (4.243, 7.508) | -0.259 | **0.008** |
| ventral part | NE | 0.168 (0.158, 0.176) | 0.174 (0.163, 0.187) | -0.247 | **0.023** |
|  | FA | 0.178 (0.173, 0.184) | 0.182 (0.175, 0.192) | -0.218 | **0.013** |

Values presented as median (IQR) unless indicated otherwise. *FDR corrected (p<0.05 in bold). Abbreviations: FA, Fractional Anisotropy; FDR, false discovery rate; HC, healthy controls; IQR, interquartile range; MDD, major depressive disorder; NB, nodal betweenness, ND; nodal degree; NE nodal efficiency; PCUN, precuneus; preCG, precentral gyrus; SFG, superior frontal gyrus; SPL, superior parietal gyrus.

**Table S5.** Associations in MDD and HC groups of AC with nodal centralities and FA metrics in brain regions which show altered AC in MDD patients

| Region | Metric | MDD (n=170) | |  | HC (n=137) | |
| --- | --- | --- | --- | --- | --- | --- |
|  |  | r | P* |  | r | P* |
| Right SFG,  posterior part | NB | -0.09 | 0.58 |  | 0.75 | **<0.001** |
|  | ND | 0.04 | 0.62 |  | 0.93 | **<0.001** |
|  | NE | 0.06 | 0.58 |  | 0.67 | **<0.001** |
|  | FA | 0.06 | 0.58 |  | -0.12 | 0.27 |
| Right SFG,  dorsolateral part | NB | 0.13 | 0.09 |  | 0.84 | **<0.001** |
|  | ND | 0.18 | **0.03** |  | 0.95 | **<0.001** |
|  | NE | 0.18 | **0.03** |  | 0.73 | **<0.001** |
|  | FA | 0.18 | **0.03** |  | 0.23 | 0.06 |
| Right preCG | NB | 0.14 | 0.10 |  | 0.76 | **<0.001** |
|  | ND | 0.24 | **<0.01** |  | 0.90 | **<0.001** |
|  | NE | 0.20 | **0.02** |  | 0.75 | **<0.001** |
|  | FA | 0.12 | 0.12 |  | 0.07 | 0.56 |
| Left SPL,  dorsal part | NB | -0.04 | 0.59 |  | 0.57 | **<0.001** |
|  | ND | 0.25 | **<0.01** |  | 0.91 | **<0.001** |
|  | NE | 0.25 | **<0.01** |  | 0.49 | **<0.001** |
|  | FA | -0.05 | 0.59 |  | -0.04 | 0.69 |
| Left SPL,  posterior part | NB | 0.19 | **0.02** |  | 0.45 | **<0.001** |
|  | ND | 0.44 | **<0.001** |  | 0.88 | **<0.001** |
|  | NE | 0.35 | **<0.001** |  | 0.38 | **<0.001** |
|  | FA | -0.21 | **<0.01** |  | -0.06 | 0.59 |
| Left PCUN,  dorsal part | NB | 0.08 | 0.31 |  | 0.41 | **<0.001** |
|  | ND | 0.26 | **<0.01** |  | 0.89 | **<0.001** |
|  | NE | 0.29 | **<0.001** |  | 0.31 | **<0.001** |
|  | FA | 0.16 | **0.04** |  | 0.17 | 0.12 |
| Left PCUN,  ventral part | NB | 0.11 | 0.18 |  | 0.42 | **<0.001** |
|  | ND | 0.15 | 0.09 |  | 0.89 | **<0.001** |
|  | NE | 0.15 | 0.09 |  | 0.30 | **<0.001** |
|  | FA | 0.21 | **0.02** |  | 0.19 | 0.09 |

*FDR corrected (p<0.05 in bold). Abbreviations: FA, Fractional Anisotropy; HC, healthy controls; MDD, major depressive disorder; NB, nodal betweenness, ND; nodal degree; NE nodal efficiency; PCUN, precuneus; preCG, precentral gyrus; SFG, superior frontal gyrus; SPL, superior parietal gyrus.

**References**

Achard, S., & Bullmore, E. (2007). Efficiency and cost of economical brain functional networks. *PLoS Comput Biol, 3*(2), e17. doi:10.1371/journal.pcbi.0030017

Cammoun, L., Gigandet, X., Meskaldji, D., Thiran, J. P., Sporns, O., Do, K. Q., . . . Hagmann, P. (2012). Mapping the human connectome at multiple scales with diffusion spectrum MRI. *J Neurosci Methods, 203*(2), 386-397. doi:10.1016/j.jneumeth.2011.09.031

Hagmann, P., Cammoun, L., Gigandet, X., Meuli, R., Honey, C. J., Wedeen, V. J., & Sporns, O. (2008). Mapping the structural core of human cerebral cortex. *PLoS Biol, 6*(7), e159. doi:10.1371/journal.pbio.0060159

Rubinov, M., & Sporns, O. (2010). Complex network measures of brain connectivity: uses and interpretations. *Neuroimage, 52*(3), 1059-1069. doi:10.1016/j.neuroimage.2009.10.003

Wang, J., Wang, X., Xia, M., Liao, X., Evans, A., & He, Y. (2015). GRETNA: a graph theoretical network analysis toolbox for imaging connectomics. *Front Hum Neurosci, 9*, 386. doi:10.3389/fnhum.2015.00386

Watts, D. J., & Strogatz, S. H. (1998). Collective dynamics of 'small-world' networks. *Nature, 393*(6684), 440-442. doi:10.1038/30918

Zhang, J., Wang, J., Wu, Q., Kuang, W., Huang, X., He, Y., & Gong, Q. (2011). Disrupted brain connectivity networks in drug-naive, first-episode major depressive disorder. *Biol Psychiatry, 70*(4), 334-342. doi:10.1016/j.biopsych.2011.05.018
